# Vunakizumab-induced severe ulcerative colitis during treatment of psoriatic arthritis: a case report and literature review

**DOI:** 10.3389/fimmu.2026.1904435

**Published:** 2026-09-04

**Authors:** Yanan Cui, Jianguo Tian, Zhenzhen Ma

**Affiliations:** 1 Department of Rheumatology and Immunology, Shengli Oilfield Central Hospital, Dongying, Shandong, China; 2 Department of Medical Imaging, Shengli Oilfield Central Hospital, Dongying, Shandong, China

**Keywords:** psoriatic arthritis, ulcerative colitis, vunakizumab, IL-17A inhibitor, drug-induced colitis, hepatic injury, biologic switching, guselkumab

## Abstract

**Background:**

Interleukin-17A (IL-17A) inhibitors are effective therapies for psoriasis (PsO) and psoriatic arthritis (PsA), yet they may induce or exacerbate inflammatory bowel disease (IBD). Vunakizumab is a novel humanized anti-IL-17A monoclonal antibody approved in China in 2024; post-marketing real-world data on its gastrointestinal safety profile remain limited.

**Case presentation:**

We report a 54-year-old male PsA patient who developed acute severe ulcerative colitis (UC) approximately one week after the third injection of vunakizumab, representing the first reported case of vunakizumab-associated IBD in the literature. The patient exhibited marked hepatic enzyme elevation (alanine aminotransferase [ALT] 213 U/L, aspartate aminotransferase [AST] 189 U/L), representing one of the most significant liver injuries reported in this context. The Naranjo Adverse Drug Reaction Probability Scale score was 7 (“probable”). Vunakizumab was immediately discontinued; intravenous methylprednisolone 60 mg/day was administered to induce remission. Following acute-phase control, the patient was switched to guselkumab (an anti-IL-23p19 monoclonal antibody) for maintenance therapy, achieving dual remission of intestinal and articular manifestations.

**Conclusion:**

Vunakizumab can induce severe UC. As a newly approved IL-17A inhibitor in China, early post-marketing safety surveillance should remain vigilant for IBD risk. The underlying mechanism may involve disruption of the intestinal epithelial barrier and compensatory upregulation of IL-23 following IL-17A blockade. The early treatment period (within the first 3 months) represents a high-risk window. Upon diagnosis, immediate drug withdrawal, corticosteroid-induced remission, and prioritized conversion to an IL-23 inhibitor (such as guselkumab) may be considered as a potential option to achieve dual control of intestinal, skin, and joint disease.

## 1 Introduction

Psoriatic arthritis (PsA) is an immune-mediated chronic inflammatory disease characterized by skin psoriasis and peripheral and axial joint inflammation. The IL-23/IL-17A immune axis plays a pivotal role in the pathogenesis of PsA and psoriasis (PsO), with IL-17A serving as the key effector cytokine driving synovial and cutaneous inflammation (1). Vunakizumab is a novel humanized IgG1 monoclonal antibody that neutralizes IL-17A with high affinity; it was approved in China in 2024 for the treatment of moderate-to-severe plaque psoriasis and ankylosing spondylitis. However, as IL-17A inhibitors have been increasingly used in real-world practice, their gastrointestinal safety concerns have gradually attracted attention.

IL-17A plays a complex and paradoxical role in intestinal mucosal immunity: on one hand, it participates in maintaining intestinal epithelial barrier integrity and antimicrobial defense; on the other hand, excessive blockade of IL-17A may disrupt intestinal barrier homeostasis and induce or exacerbate IBD (2, 3). Clinical trials have confirmed that IL-17A inhibitors are not only ineffective for Crohn’s disease (CD) but may also worsen disease activity (4, 5). In randomized controlled trials and post-marketing surveillance of patients with PsO, PsA, and ankylosing spondylitis, case reports of new-onset IBD or acute exacerbation of pre-existing IBD have emerged (6–8).

More recently, a systematic review and meta-analysis by Zhang et al. (9) analyzed 21 RCTs (n=16,557) comparing the risk of new-onset IBD among five interleukin inhibitors in psoriasis patients. Notably, only ixekizumab was significantly associated with an increased IBD risk (MH RD 0.0027, 95%CI 0.0001–0.0054, P = 0.04), whereas secukinumab, bimekizumab, brodalumab, and ustekinumab showed no significant difference compared with controls. Vunakizumab was not included in this analysis, as it was approved in China in 2024 after the study’s search cutoff. In a comprehensive review of vunakizumab, Yan et al. (10) reported that during the 52-week phase III psoriasis trial (n=460), one case of moderate Crohn’s disease occurred during long-term exposure, and hepatic enzyme elevations were observed in 11.1% of patients by week 52. These findings suggest that the IBD risk may vary across IL-17A inhibitors and that post-marketing surveillance of vunakizumab is warranted.

Long-term safety data for vunakizumab, particularly regarding IBD risk in real-world studies, remain scarce. Herein, we report a 54-year-old male PsA patient who developed acute severe UC approximately one week after the third injection of vunakizumab (approximately 5 weeks after treatment initiation). Following drug withdrawal, corticosteroid-induced remission, and conversion to guselkumab (an anti-IL-23p19 monoclonal antibody), the patient achieved a favorable outcome. This case suggests a potential safety signal for post-marketing surveillance of this newly approved agent in China, and we systematically review the mechanisms, clinical characteristics, and reported management strategies of IL-17A inhibitor-associated IBD.

## 2 Case presentation

**Figure 1:**
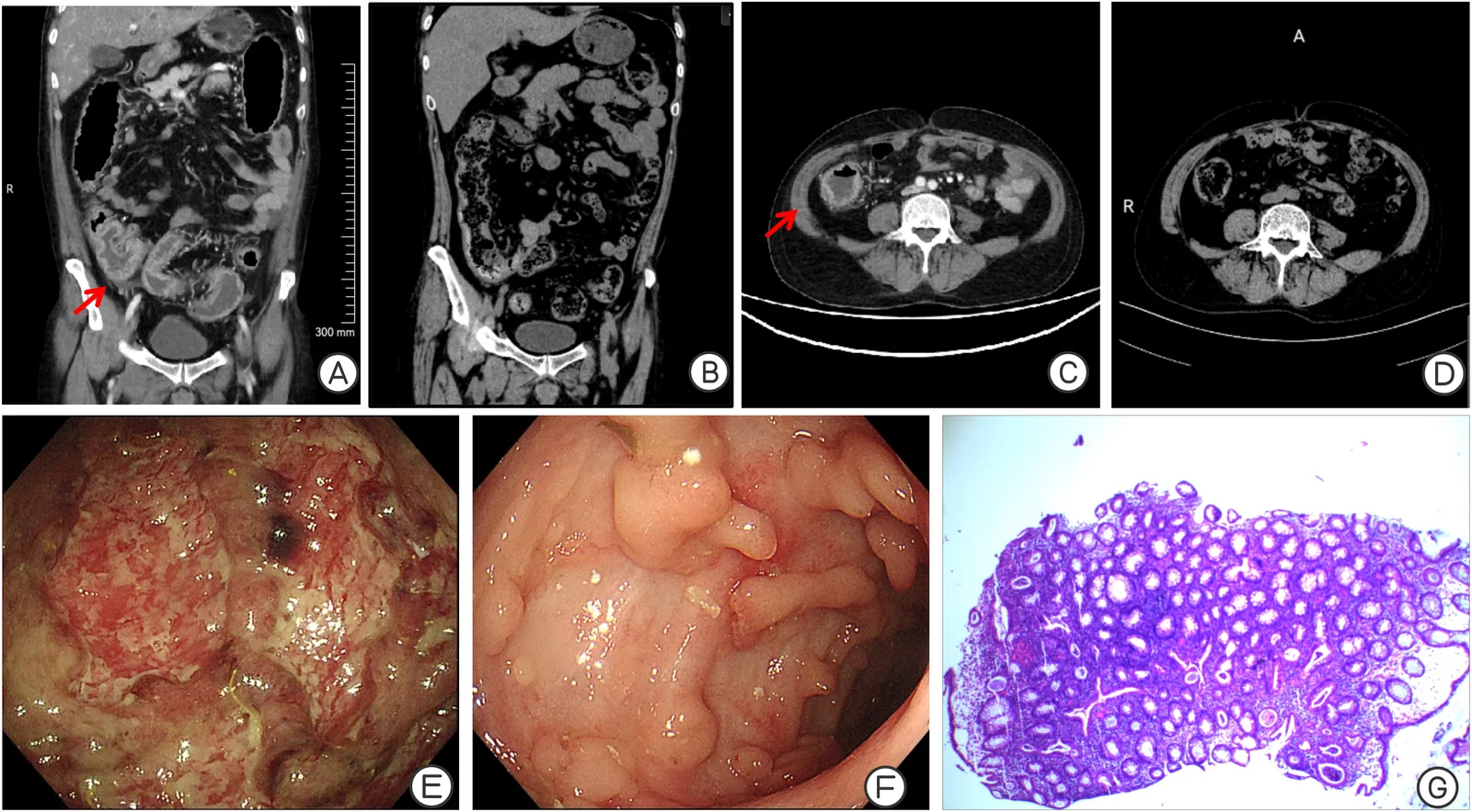
Imaging, endoscopic, and histopathological findings. **(A, C)** Contrast-enhanced abdominal CT at baseline showing extensive asymmetric bowel wall thickening and enhancement with luminal narrowing and pericecal exudate (arrows). **(B, D)** Follow-up abdominal CT after treatment showing reduced colonic distension, decreased bowel wall thickening, and diminished perienteric exudate. **(E)** Sigmoidoscopy at baseline showing diffuse deep large ulcers with marked hyperemia and edema, yellow-white exudate, and friable mucosa prone to bleeding. **(F)** Follow-up colonoscopy showing multiple ulcer scars and mucosal hyperplasia throughout the colon. **(G)** Rectal mucosal biopsy showing chronic inflammation with mucosal erosion, diffuse interstitial lymphocytic and plasmacytic infiltration, crypt architectural distortion, cryptitis, and crypt abscesses (H&E, ×100).

A 54-year-old male patient was admitted on February 4, 2026, with a 20-day history of abdominal pain accompanied by purulent bloody stool. He was diagnosed with psoriasis and PsA in 2006 and had previously received methotrexate (10 mg/week) and adalimumab (40 mg every 2 weeks), both of which were discontinued due to inadequate joint and skin control. No family history of IBD or autoimmune diseases was reported. Beginning in December 2025, he received subcutaneous vunakizumab 240 mg every 2 weeks for a total of three doses; the last dose was administered on January 28, 2026. Prior to initiating vunakizumab, the patient had no gastrointestinal symptoms (no abdominal pain, diarrhea, hematochezia, or tenesmus). No baseline gastrointestinal evaluation, including fecal calprotectin testing or colonoscopy, was performed, as there were no clinical indications at that time. On February 4, 2026 (day 7 after the last injection), he developed sudden periumbilical and lower abdominal pain accompanied by diarrhea (>10 times/day), purulent bloody stool, tenesmus, and fever (maximum temperature 38.7 °C). At a local hospital, colonoscopy revealed multiple colonic ulcers with bleeding, consistent with severe active colitis. He was treated with oral mesalazine (dose unknown) and supportive care, but symptoms progressively worsened. Laboratory tests at the local hospital showed C-reactive protein (CRP) 102.32 mg/L and erythrocyte sedimentation rate (ESR) 75 mm/h. Contrast-enhanced abdominal computed tomography (CT) revealed thickening of the rectal and colonic walls with surrounding exudate, suggestive of inflammatory changes (**Figures 1A, C**). Despite anti-infective therapy, acid suppression, and electrolyte correction, his condition failed to improve, and he was transferred to our hospital on February 24, 2026.

On admission, physical examination revealed a temperature of 38.3 °C, pulse 105 beats/min, respiration 19 breaths/min, and blood pressure 103/69 mmHg. The abdomen was flat and soft, with tenderness in the lower abdomen but no rebound tenderness or muscle guarding. Laboratory investigations were as follows: Complete blood count: white blood cell count (WBC) 10.98 × 10^9^/L, hemoglobin (Hb) 91 g/L, platelet count (PLT) 183 × 10^9^/L; Inflammatory markers: CRP 128.66 mg/L, ESR 36 mm/h; Liver function: ALT 213 U/L, AST 189 U/L, gamma-glutamyl transferase (GGT) 113 U/L, albumin (ALB) 16.5 g/L, direct bilirubin (DBiL) 7.1 μmol/L; Immunology: HLA-B27, rheumatoid factor (RF), anti-cyclic citrullinated peptide (CCP) antibody, antineutrophil cytoplasmic antibodies (ANCA), and antinuclear antibody profiles were all negative; Microbiology: stool culture, clostridioides difficile toxin, and cytomegalovirus (CMV) nucleic acid testing were negative.

Colonoscopy revealed: at 30 cm from the anal verge (sigmoid-descending colon junction), diffuse deep large ulcers were observed with mucosal hyperemia, edema, yellow-white exudate, and friable mucosa prone to bleeding (**Figure 1E**); at 12–15 cm from the anal verge (rectum), multiple focal deep punched-out ulcers with white exudate and obscured submucosal vessels were seen. Pathology of the rectal biopsy showed chronic inflammation with mucosal erosion, diffuse interstitial lymphocytic and plasmacytic infiltration, crypt architectural distortion, cryptitis, and crypt abscesses (**Figure 1G**). Sigmoid colon biopsy showed inflammatory granulation tissue and exudate. Immunohistochemistry was negative for CMV and EBER; acid-fast bacillus (AFB) staining was negative.

Diagnosis: Ulcerative colitis (severe active phase, fulfilling Truelove-Witts criteria: stool frequency >6/day, temperature >37.8 °C, pulse >90/min, Hb <105 g/L, ESR >30 mm/h);, PsA, severe hypoalbuminemia, elevated hepatic enzymes, and electrolyte disturbance.

**Figure 2:**
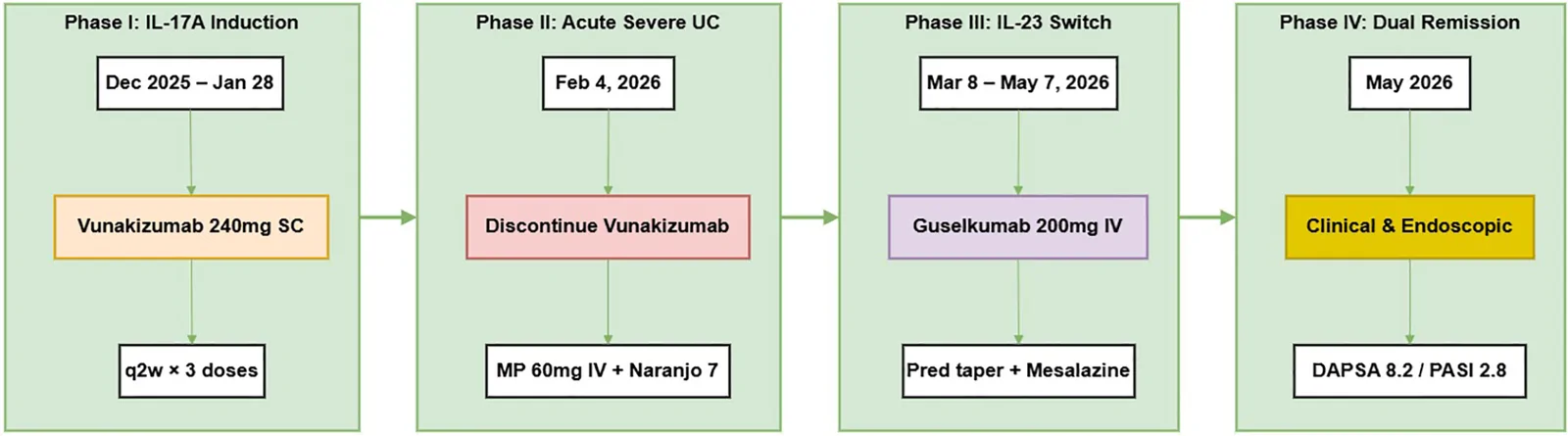
Treatment timeline of the present case.

Treatment: The treatment timeline is illustrated in **Figure 2**. Following rheumatology consultation, a clear temporal relationship between vunakizumab and UC onset was established. The Naranjo Adverse Drug Reaction Probability Scale score was 7 (“probable”; **Supplementary Table 1**). Vunakizumab was discontinued immediately. The patient received intravenous methylprednisolone 60 mg/day for anti-inflammatory therapy, ceftriaxone for infection prophylaxis, magnesium isoglycyrrhizinate for hepatoprotection, human albumin for hypoalbuminemia correction, probiotics for intestinal flora modulation, and parenteral nutritional support. After 8 days of treatment with intravenous methylprednisolone (March 8, 2026), diarrhea and fever resolved, inflammatory markers declined, and the patient was deemed to have achieved a good steroid response. Methylprednisolone was tapered to 40 mg/day, and guselkumab 200 mg intravenous infusion was initiated (week 0). At discharge (March 15, 2026), the patient was prescribed oral prednisone 45 mg/day (planned taper: 5 mg/week reduction to 25 mg/day, then 5 mg every 2 weeks until discontinuation) and mesalazine enteric-coated tablets 2 g twice daily. Guselkumab 200 mg intravenous infusions were administered at week 4 (April 8, 2026) and week 8 (May 7, 2026).

At follow-up (May 2, 2026, 5 days before the week 8 guselkumab infusion), repeat abdominal CT showed reduced colonic distension, decreased bowel wall thickening, and diminished perienteric exudate compared with the previous scan (**Figures 1B, D**). On May 6, 2026 (1 day before the week 8 guselkumab infusion), repeat colonoscopy showed: the ascending and transverse colon exhibited lead-pipe appearance with multiple ulcer scars and mucosal hyperplasia; the descending and sigmoid colon mucosa was relatively smooth with multiple ulcer scars and mucosal hyperplasia; the rectosigmoid junction showed mild luminal narrowing with mucosal edema, scattered ulcers covered with white exudate, and multiple white ulcer scars with mucosal hyperplasia; the rectal mucosa was smooth with scattered mucosal hyperplasia (**Figure 1F**). The patient reported no abdominal pain or diarrhea. PsA disease activity assessed by the Disease Activity in Psoriatic Arthritis (DAPSA) score decreased from a baseline of 24.6 to 8.2 (low disease activity), and the Psoriasis Area and Severity Index (PASI) decreased from 12.5 to 2.8. Both PsA and UC were well controlled.

Long-term follow-up: The patient found the treatment regimen acceptable and complied with the protocol throughout the study. At the 4-month follow-up on July 15, 2026, the patient remained asymptomatic with no abdominal pain, diarrhea, hematochezia, fever, or arthralgia. Inflammatory markers were completely normalized (ESR and CRP within normal ranges). The patient had successfully completed corticosteroid tapering and discontinuation approximately 3 weeks prior. He continued maintenance therapy with mesalazine enteric-coated tablets 2 g twice daily and received the fourth dose of guselkumab 100 mg subcutaneous injection. No recurrence of ulcerative colitis was observed.

## 3 Discussion

### 3.1 Role of IL-17A in intestinal immunity and the mechanism of IBD induction

**Figure 3:**
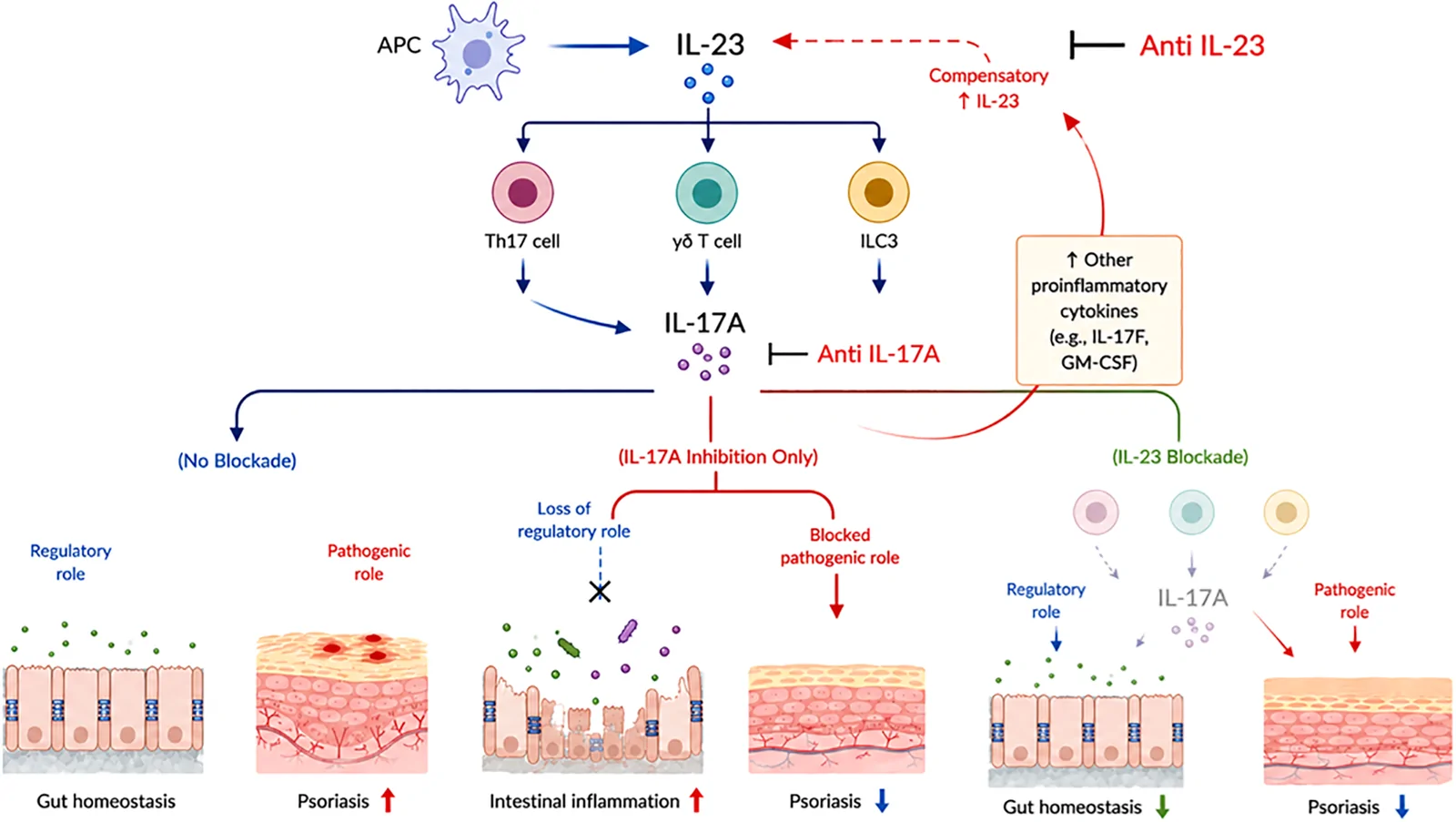
Under homeostasis, IL-17A (derived from Th17, γδ T, and ILC3 cells) preserves intestinal barrier integrity by regulating tight junctions, whereas IL-23 promotes IL-17A production. Although IL-17A blockade ameliorates psoriasis, it compromises gut homeostasis, inducing compensatory IL-23 upregulation that enhances IL-17F and GM-CSF secretion by IL-23-responsive cells, thereby aggravating intestinal inflammation. Conversely, IL-23 inhibition suppresses upstream immune activation, reducing IL-17A and downstream inflammatory cytokines to improve both intestinal and cutaneous inflammation. APC, antigen-presenting cell; ILC3, group 3 innate lymphoid cell; GM-CSF, granulocyte-macrophage colony-stimulating factor.

IL-17A is primarily produced by Th17 cells, γδ T cells, and group 3 innate lymphoid cells (ILC3), and serves as a core effector cytokine in the pathogenesis of PsO, PsA, and ankylosing spondylitis. Targeting IL-17A can rapidly and effectively control skin and joint inflammation. However, the role of IL-17A in the intestinal microenvironment is far more complex than in the skin, and it plays an important protective role for the intestinal mucosal barrier. First, IL-17A participates in maintaining intestinal epithelial barrier integrity. Lee et al. (2) demonstrated that γδ T cells in the intestinal lamina propria are an important source of IL-17A; the IL-17A produced regulates tight junction protein localization, thereby maintaining inter-epithelial tight junctions and limiting mucosal permeability. In acute intestinal injury mouse models, neutralization or knockout of IL-17A increased intestinal permeability, bacterial translocation, and inflammation. Therefore, blockade of the IL-17A pathway may disrupt intestinal barrier function and trigger acute UC onset. In addition to its role in barrier maintenance, IL-17A induces epithelial cells to produce antimicrobial peptides (e.g., β-defensins, S100 proteins) and proinflammatory chemokines (e.g., CXCL1, CCL20) (11, 12). Deep blockade of these protective and regulatory functions by IL-17A inhibitors may disrupt the delicate balance between host defense and inflammation in the intestinal mucosa, potentially rendering the gut more susceptible to microbial dysbiosis and inflammatory injury. The paradoxical role of IL-17A in intestinal immunity and the rationale for switching to IL-23 inhibition are illustrated in **Figure 3**. Second, IL-17A exerts negative feedback regulation on the IL-23/Th17 axis. IL-23 lies upstream of this pathway and drives Th17 cell proliferation and differentiation. Studies have shown that IL-17A can feedback-inhibit IL-23 production by dendritic cells and macrophages; anti-IL-17A therapy may relieve this negative feedback, leading to compensatory IL-23 overexpression and thereby exacerbating Th17-mediated inflammation (3). This explains why IL-23 inhibitors are effective for IBD, whereas IL-17A inhibitors are ineffective or even worsen the disease. In the present case, conversion from an IL-17A inhibitor to an IL-23 inhibitor resulted in clinical remission, corroborating this theory from a clinical perspective.

### 3.2 Differential IBD risk across IL-17A inhibitors

The risk of IBD induction may vary across IL-17A inhibitors due to differences in molecular structure and pharmacokinetic properties. Vunakizumab is a humanized IgG1/κ monoclonal antibody with a terminal half-life of approximately 24 days and steady-state trough concentrations of ~28.7 μg/mL (240 mg Q4W) (10). In contrast, ixekizumab is a modified humanized IgG4 antibody with a shorter half-life (~13 days) and lower steady-state concentrations (~3.5 μg/mL at Q4W maintenance), whereas secukinumab is a fully human IgG1 with a comparable half-life (22–31 days) but different dosing regimen (10). These pharmacokinetic differences may influence the depth and duration of intestinal IL-17A blockade. Notably, Zhang et al. (9) identified ixekizumab as the only IL-17A inhibitor with a statistically significant association with IBD risk in their meta-analysis, despite its shorter half-life, suggesting that factors beyond pharmacokinetics—such as immunogenicity (ixekizumab ADA ~22% at week 60 vs. vunakizumab ~5% at week 52) or tissue distribution—may also contribute (10). In the vunakizumab clinical program, one case of moderate Crohn’s disease was reported during long-term exposure (10). The present case of severe UC with marked hepatic injury expands this limited safety dataset and underscores the need for head-to-head comparative studies to clarify whether structural and pharmacokinetic differences translate into differential IBD risk.

### 3.3 Clinical features of IL-17A inhibitor-associated IBD and comparison with previous cases

Since the advent of IL-17A inhibitors, multiple case reports and clinical studies have confirmed their association with IBD. We systematically searched PubMed, Embase, Cochrane Library, and CNKI using the search terms (“IL-17 inhibitor” OR “secukinumab” OR “ixekizumab” OR “vunakizumab” OR “brodalumab”) AND (“inflammatory bowel disease” OR “Crohn’s disease” OR “ulcerative colitis”), with a cutoff date of May 31, 2026. A total of 14 cases of IL-17A inhibitor-induced IBD were identified (10 with secukinumab, 4 with ixekizumab). Analysis of these 14 cases from 13 published reports plus the present case (n = 15; **Supplementary Table 2**) revealed the following characteristics (13–25):

1. Sex and age distribution: Among the 15 cases, 8 were male and 7 were female, showing a balanced sex distribution. Ages ranged from 19 to 65 years, with a median age of 41.5 years (interquartile range [IQR] 35.75–44.25 years). The present patient was a 54-year-old male, outside the common age range.
2. Underlying disease and IL-17A inhibitor type: The underlying diseases were predominantly psoriasis (including PsO and PsA) in 14 cases, with one case of SAPHO syndrome; one PsA patient also had ankylosing spondylitis. The drugs involved included secukinumab (10 cases), ixekizumab (4 cases), and vunakizumab (1 case, the present case, representing the first reported case in the literature).
3. IBD type and severity: Both UC (7 cases) and CD (6 cases) were reported, with 2 cases unclassified. Notably, one patient with severe UC and one with CD developed toxic megacolon and ultimately underwent total colectomy (13, 14). This suggests that IL-17A inhibitor-induced IBD may be highly aggressive, underscoring the importance of early recognition and intervention.
4. Time to onset: The interval from treatment initiation to symptom onset ranged from 1 week to 56 months; 12 cases (80.0%) developed IBD within 6 months. Wang et al. (15) and Morosanu et al. (13) reported onset at 1 week, while Rodríguez Moncada et al. (16) reported onset at 3 weeks. The present patient developed symptoms approximately one week after the third injection (approximately 5 weeks after treatment initiation). Yamada et al. (7) meta-analysis showed that approximately 25% of IBD events occurred within the first 3 months of treatment, suggesting that IL-17A inhibitor-induced intestinal barrier injury is an early, direct effect.
5. Family history: Among the 15 cases, only 4 had a definite family history of IBD; the present patient had no such history. Although family history is an important risk factor, the majority of drug-induced IBD cases occurred in individuals without a known family history; therefore, risk stratification should not rely solely on family history.
6. Hepatic injury: The incidence and mechanism of hepatic injury in IL-17A inhibitor-associated IBD have not received sufficient attention. Among the 15 cases, 3 (20.0%) exhibited abnormal liver function, but previous reports mostly described mild enzyme elevation (ALT <100 U/L). In the present case, ALT was 213 U/L and AST was 189 U/L, representing one of the most significant hepatic enzyme elevations reported to date. This suggests that hepatic injury may be an underestimated but important accompanying event of this adverse reaction. The mechanism may be related to the following factors: (i) disruption of the intestinal barrier and bacterial translocation following IL-17A blockade, leading to endotoxemia and activation of hepatic Kupffer cells; (ii) hepatocyte injury caused by systemic inflammatory storm; (iii) the longer half-life of vunakizumab compared with other IL-17A inhibitors may lead to sustained drug exposure in hepatic tissue, and its high-affinity deep blockade of IL-17A may continuously activate Kupffer cells and amplify systemic inflammatory loops, thereby exacerbating liver injury. This hypothesis awaits further validation by pharmacokinetic-pharmacodynamic (PK-PD) studies and direct comparative hepatotoxicity data in larger cohorts. Whether the observed hepatic enzyme elevation represents a drug-specific adverse effect of vunakizumab rather than a consequence of severe IBD itself cannot be definitively distinguished in this single case. Regardless of the precise mechanism, the present case highlights that hepatic enzyme elevation (ALT, AST) may accompany IL-17A inhibitor-associated IBD and should be monitored routinely in addition to gastrointestinal symptoms, particularly during the initial treatment period (first 3 months).
7. Treatment switching and prognosis: All cases discontinued the IL-17A inhibitor. The vast majority (14/15) received corticosteroid therapy to induce remission. Subsequent biologic switching strategies: 9 patients initially received anti-TNF-α agents (infliximab, adalimumab, golimumab, certolizumab pegol), of whom 3 were subsequently switched to IL-12/23 or IL-23 inhibitors (ustekinumab, risankizumab) due to inadequate control of the underlying disease or IBD relapse; 5 patients were directly switched to IL-12/23 or IL-23 inhibitors (ustekinumab, guselkumab, risankizumab, mirikizumab, tildrakizumab), all achieving remission. In the present case, direct conversion to guselkumab after steroid-induced remission was associated with good control of both PsA and UC at the current follow-up. At the 4-month follow-up, the patient remained in dual remission with normalized inflammatory markers and no recurrence of UC, supporting the durability of this treatment strategy in this case. Ishida et al. (17) reported UC induced by secukinumab that remitted following mirikizumab (anti-IL-23p19) therapy. Existing evidence from case reports and small series suggests that for IL-17A inhibitor-induced IBD, direct switching to an IL-23 inhibitor may represent a reasonable alternative to anti-TNF-α therapy, though comparative data from randomized trials are lacking.

### 3.4 Differential diagnosis and causality assessment

The diagnosis of the present case underwent rigorous differential diagnosis, primarily excluding the following conditions: (1) Infectious colitis: Stool culture, clostridioides difficile toxin, cytomegalovirus (CMV) DNA, and Epstein-Barr virus-encoded RNA (EBER) in pathology were all negative. The patient had no history of recent travel, consumption of contaminated food, or antibiotic use prior to symptom onset. Empiric anti-infective therapy administered at the local hospital failed to improve symptoms, rendering infectious etiology highly unlikely. (2) Ischemic colitis: The patient had no underlying cardiovascular disease (no hypertension, diabetes mellitus, atrial fibrillation, or atherosclerosis), no history of hypotension or shock, and no use of vasoactive medications. Contrast-enhanced abdominal CT showed no mesenteric vascular stenosis, thrombosis, or embolism. Furthermore, the endoscopic distribution of lesions (diffuse involvement from rectum to sigmoid-descending colon junction) did not correspond to typical vascular territories (e.g., watershed areas such as the splenic flexure), making ischemic colitis improbable. (3) NSAID-associated enteropathy: The patient denied long-term or high-dose nonsteroidal anti-inflammatory drug (NSAID) use. Endoscopic findings of diffuse deep large ulcers with crypt architectural distortion, cryptitis, and crypt abscesses are characteristic of inflammatory bowel disease rather than NSAID enteropathy, which typically presents with diaphragm-like strictures, web-like ulcers, or small intestinal involvement. (4) Radiation enterocolitis: The patient had no history of abdominal or pelvic radiation therapy, excluding this diagnosis. (5) Vasculitis-associated colitis: Serological testing for antineutrophil cytoplasmic antibodies (ANCA), antinuclear antibodies (ANA), and other autoimmune markers was negative. The patient exhibited no systemic manifestations of vasculitis (no skin purpura, renal involvement, or peripheral neuropathy), making vasculitic colitis unlikely. (6) *De novo* IBD comorbid with psoriasis: psoriasis and IBD share genetic susceptibility loci such as IL23R and STAT3 (26), and their co-occurrence is higher than in the general population. However, several features in the present case argue against *de novo* IBD: (i) the patient had a 20-year history of psoriasis and psoriatic arthritis without any gastrointestinal symptoms; (ii) if subclinical IBD had been present, it would typically manifest as mild proctitis or mildly active disease, whereas the present case showed acute-onset severe pancolitis-type ulcerative colitis with deep large ulcers and a risk of toxic megacolon; (iii) the temporal relationship between drug exposure and symptom onset was clear (symptoms developed approximately one week after the third injection, approximately five weeks after treatment initiation); and (iv) the Naranjo score indicated a “probable” causal relationship (see below). Nevertheless, we acknowledge that pre-treatment colonoscopic screening was not performed, so the possibility of unmasking subclinical IBD cannot be completely excluded theoretically. (7) Causality assessment: The causal relationship between vunakizumab and ulcerative colitis was formally evaluated using the Naranjo Adverse Drug Reaction Probability Scale. The present case scored 7 points, classified as a “probable” adverse drug reaction (scores ≥9: definite; 5–8: probable; 1–4: possible; ≤0: doubtful). The detailed scoring is presented in Supplementary **Table 1**. Key scoring items included: (i) previous conclusive reports on this reaction exist in the literature (+1 point); IL-17A inhibitors including secukinumab and ixekizumab have been reported to induce or exacerbate inflammatory bowel disease in multiple case reports and clinical studies (6–8, 15); (ii) the adverse event appeared after the suspected drug was administered (+2 points); (iii) the adverse event improved when the drug was discontinued (+1 point); (iv) the adverse event reappeared when the drug was readministered—this item was scored 0 as rechallenge was not performed; (v) there were no alternative causes that could on their own have caused the reaction (+2 points), as infectious, ischemic, NSAID-associated, radiation, and vasculitic etiologies were systematically excluded (see above); (vi) the reaction did not reappear when a placebo was given—this item was scored 0 as no placebo was administered; (vii) the drug was not detected in blood at toxic concentrations—this item was scored 0 as drug levels were not measured; (viii) the reaction was not more severe with higher dose or less severe with lower dose—this item was scored 0 as dose escalation was not performed; (ix) the patient had no similar reaction to the same or similar drugs in the past—this item was scored 0 as no prior exposure to IL-17A inhibitors; and (x) the adverse event was confirmed by objective evidence (+1 point), including colonoscopy, histopathology, and contrast-enhanced abdominal CT. The detailed scoring is presented in **Supplementary Table 1**.

### 3.5 Treatment strategy: switching from IL-17A inhibition to IL-23 inhibition

Upon diagnosis, vunakizumab was immediately discontinued, and intravenous methylprednisolone 60 mg/day was administered to induce remission, concurrently with fasting, gastrointestinal decompression, correction of hypoalbuminemia and electrolyte disturbances, infection control, and comprehensive supportive care. After acute-phase control, given the need for long-term biologic therapy for PsA and the clear IBD induction and significant hepatic injury associated with IL-17A blockade, the patient was switched to guselkumab (targeting the IL-23 p19 subunit). Diarrhea and fever gradually resolved, intestinal inflammation was well controlled, and psoriatic skin and joint symptoms improved simultaneously.

This outcome carries important implications: the inflammatory drive in IL-17A inhibitor-associated IBD appears to be closely related to the IL-23/Th17 axis. Switching from targeting IL-17A (a downstream effector) to blocking IL-23 (an upstream regulator) may represent a mechanistic shift from “downstream effector blockade” to “upstream regulator blockade,” potentially avoiding the intestinal barrier imbalance caused by IL-17A inhibition while maintaining immunomodulation for PsA (13). Guselkumab, a selective IL-23 inhibitor, has established efficacy in PsA, and existing evidence indicates that it does not increase IBD risk (14). Unlike some reports in the literature describing prolonged symptoms after IL-17A inhibitor withdrawal or the need for anti-TNF-α therapy, the present case achieved dual control of intestinal and joint disease through rapid steroid-induced remission and guselkumab maintenance. Whether this outcome is reproducible in other patients remains to be determined.

### 3.6 Clinical implications and limitations

This case suggests that when using IL-17A inhibitors such as vunakizumab for psoriasis and PsA, clinicians should: (1) Pre-treatment screening: Obtain a detailed history of IBD, family history, and unexplained gastrointestinal symptoms; perform fecal calprotectin testing or colonoscopy when indicated. For high-risk patients with concurrent psoriasis and IBD, these agents should be used cautiously or avoided. In the present case, the patient had no gastrointestinal symptoms before starting vunakizumab, and no baseline fecal calprotectin testing or colonoscopy was performed, as there were no clinical indications at that time. However, in retrospect, such baseline screening might have helped exclude subclinical disease and should be considered in future clinical practice, particularly for patients with any subtle gastrointestinal complaints. (2) On-treatment monitoring: Given the differential IBD risk across IL-17A inhibitors and the emerging safety data for vunakizumab, clinicians should maintain heightened vigilance during the initial treatment period (first 3 months), which represents a high-risk window. Routinely inquire about gastrointestinal symptoms; if abdominal pain, diarrhea, or hematochezia occur, fecal calprotectin testing and colonoscopy should be promptly performed. Liver function (ALT, AST) should be monitored concurrently, as hepatic enzyme elevations have been reported in 11.1% of patients by week 52 in clinical trials and may serve as an early accompanying signal of this adverse reaction (10). Patients with rapidly worsening symptoms should be monitored for severe complications such as toxic megacolon. (3) Multidisciplinary collaboration: Once drug-related IBD is suspected, joint evaluation by gastroenterology, pathology, and radiology is warranted. For severe patients, systemic corticosteroids should be promptly initiated to induce remission, and conversion to IL-23 inhibitors with better intestinal safety profiles should be considered for maintenance.

This is a single-center case report lacking in-depth mechanistic exploration such as dynamic intestinal microbiota monitoring, mucosal immune cell phenotyping, and cytokine profiling. The follow-up duration is currently 5 months from diagnosis, with the most recent evaluation on July 15, 2026, showing sustained remission of both UC and PsA. Longer-term follow-up is ongoing to monitor the durability of guselkumab efficacy and the risk of UC relapse. In addition, the patient did not undergo pre-treatment colonoscopic screening or fecal calprotectin testing. Although the Naranjo score indicated a ‘probable’ causal relationship and alternative etiologies were systematically excluded, the possibility of unmasking subclinical IBD cannot be completely excluded theoretically. The treatment recommendation to switch directly to guselkumab is based solely on this single case report. No randomized controlled trials or comparative studies have evaluated the optimal biologic switching strategy for IL-17A inhibitor-induced IBD. Therefore, this recommendation should be interpreted with caution and validated in prospective studies before broader clinical application.

## 4 Conclusion

Long-term safety data for vunakizumab, particularly regarding IBD risk in real-world studies, remain limited. This article reports the first case of severe ulcerative colitis induced by vunakizumab in the literature, accompanied by significant hepatic enzyme elevation (ALT 213 U/L, one of the most significant reported to date). Following drug withdrawal, corticosteroid-induced remission, and conversion to guselkumab maintenance therapy, both intestinal and articular manifestations were well controlled. This case indicates that: (i) as a newly approved IL-17A inhibitor in China, vunakizumab requires heightened early post-marketing surveillance for IBD and hepatic injury risk; (ii) the early treatment period (first 3 months) represents a high-risk window, and simultaneous monitoring of gastrointestinal symptoms and liver function is recommended; and (iii) upon diagnosis, prompt drug withdrawal and prioritized conversion to an IL-23 inhibitor can achieve dual control of intestinal, skin, and joint disease, though this recommendation is based on limited evidence and requires further validation. This case provides an important safety signal for post-marketing surveillance of this newly approved agent in China, and systematically reviews the mechanisms, clinical characteristics, hepatic injury features, and treatment strategies of IL-17A inhibitor-associated IBD through literature review.

## Data Availability

The raw data supporting the conclusions of this article will be made available by the authors, without undue reservation.

## Supplementary material

Supplementary Table 1 Naranjo adverse drug reaction probability scale score.

Supplementary Table 2 Clinical features of case reports with gastrointestinal symptoms in IL-17 inhibitor-treated patients.
