# Supplementary material for: Vunakizumab-induced severe ulcerative colitis during treatment of psoriatic arthritis: a case report and literature review
Source: Front Immunol. 2026 Sep 4;17:1904435. doi: 10.3389/fimmu.2026.1904435 (PMC13585705; doi:10.3389/fimmu.2026.1904435)
Supplement: Supplementary file 1 [file Table1.docx]

**Table 1 Naranjo ADR probability score**

| **Question** | **Yes** | **No** | **Do Not Know** | **Score** |
| --- | --- | --- | --- | --- |
| 1.Are there previous conclusive reports on this reaction? | +1 | 0 | 0 | 1 |
| 2. Did the adverse event appear after the suspected drug was administered? | +2 | -1 | 0 | 2 |
| 3. Did the adverse event improve when the drug was discontinued or a specific antagonist was administered? | +1 | 0 | 0 | 1 |
| 4. Did the adverse event reappear when the drug was readministered? | +2 | -1 | 0 | 0 |
| 5. Are there alternative causes that could on their own have caused the reaction? | -1 | +2 | 0 | 2 |
| 6. Did the reaction reappear when a placebo was given? | -1 | +1 | 0 | 0 |
| 7. Was the drug detected in blood or other fluids in concentrations known to be toxic? | +1 | 0 | 0 | 0 |
| 8. Was the reaction more severe when the dose was increased or less severe when the dose was decreased? | +1 | 0 | 0 | 0 |
| 9. Did the patient have a similar reaction to the same or similar drugs in any previous exposure? | +1 | 0 | 0 | 0 |
| 10. Was the adverse event confirmed by any objective evidence? | +1 | 0 | 0 | 1 |
|  | **Total Score: 7** | | | |

Note: Score≥ 9 = Definitive, 5– 8 = Probable, 1– 4 = Possible, ≤0 = Doubtful.
