# Supplementary material for: Vunakizumab-induced severe ulcerative colitis during treatment of psoriatic arthritis: a case report and literature review
Source: Front Immunol. 2026 Sep 4;17:1904435. doi: 10.3389/fimmu.2026.1904435 (PMC13585705; doi:10.3389/fimmu.2026.1904435)
Supplement: Supplementary file 2 [file Table2.doc]

Table 2. Clinical features of case reports with gastrointestinal symptoms in IL-17 inhibitor-treated patients

| Reference | Published year | Sex/ age(year) | Underlying illness | IBD personal history | IBD family history | IL-17i | Discontinue IL-17i | IBD type | Symptoms | Imaging | Laboratory findings | Abnormal liver enzymes | Infection | Steroid | Alternative  drugs | Surgery | Outcome | Time after first dose when illness appeared |
| --- | --- | --- | --- | --- | --- | --- | --- | --- | --- | --- | --- | --- | --- | --- | --- | --- | --- | --- |
| FOBELO LOZANO^[19]^ | 2018 | F/19 | PsO | Unclear | ND | Secukinumab | Yes | CD | Nausea,abdominal pain | Ileo-colonoscopy,,magnetic resonance enterography | WBC↑,CRP↑ | No | No | Yes | Ustekinumab | No | Remission | 2 months |
| WANG^[15]^ | 2018 | F/41 | PsO | No | Yes (mother with CD,daughter with UC) | Secukinumab | Yes | Unclassified IBD | Abdominal pain,bloody  bowel movements,fever | Flexible sigmoidoscopy | CRP↑,ALB↓,HB↓ | ND | No | Yes | Infliximab | No | Clinically stable | 1 week |
| RODRíGUEZ^[16]^ | 2019 | M/42 | PsA | No | Yes (mother with UC) | Secukinumab | Yes | UC | Diarrhea,rectal bleeding | Colonoscopy | ND | ND | ND | Yes | [Golimumab](https://www.baidu.com/baidu.php?url=af0000KpxzUee8Wytc7VU_xP2qfhxqUkKbk8PxZkK41j6GrIdAaHAO-kD8cCgCNKeuysO7usZNiVHvvxtv48kGPFGE7EmHPCl-EW7vx0Zku82PqYcLABy85PvLNUqY30WdPCiwg2SdVzce_ywJR8PH_Tyvzrt5VLANLAc4bh8dSprYqNbFJl1R8CbsFY0rhHQGH-VXpCI_lEc4AdHgtp2unwiJnk.DY_a61PJHfY8CtbDQVrPva2ljRlBOH6Y_Lq3eCp5UBmq8e5zqZqZeO2OSWb3XWIolxUSENOv5oZJ4XWKqxqxdOg-Oej5lqglWreeOJXLqXHGln7L4SWxMyFWIuuuug3eSyG-LQWdQjPakgke2qB6.U1Yk0ZDq8ro0StiSVpyO8fKspynqn0KY5UWJs5EDEoy-Eeb0pyYqnWcd0ATqUvNsT100Iybqmh7GuZR0TA-b5Hcd0APGujYznW00UgfqnH0vnNtknjDLg1csPH7xnH0krNt1PW0k0AVG5H00TMfqP1010AFG5HDdr7tznjf0UynqnH0vnNtknjDLg1csPH7xnH0krNts0Z7spyfqn0Kkmv-b5H00ThIYmyTqn0K8my-b5H00Uh7YTjYs0APdujYYnWb1P1m1nHc0mycqn7ts0ANzu1Ys0ZKs5HcYnH04n1b1nH00UMus5H08nj0snj0snj00Ugws5H00uAwETjYs0ZFJ5H00uANv5gKW0AuY5H00TA6qn0KET1Ys0AFL5HDs0A4Y5H00TLCq0A71gv-bm1dsTv7zUidBuAw30A-bm1dcfbR0TA9YXHY0IA7zuvNY5Hm1g1KxnHRs0ZwdT1Y3PWm3PWDznjcvP1RdPjmvP1010ZF-TgfqnHT3njbvrHnYrjT1n6KGm1Ys0ZPGujd-nW9hujfvn10snjTvrj6s0AP1UHd7wRRkPWD4PWu7fH7DnDn30AkBT1Ys0A7W5HD0TA3qn0KkUgfqn0KkUgnqn0KlIjYs0AFvujYs0AIsIjYk0AI9IjYk0AdWgvuzUvYqn7tsg1Kxn7ts0Aw9UMNBuNqsUA78pyw15HRYPHcYPHmzg1Kxn7tsg100TA7Ygvu_myTqn0Kbmv-b5HnkPjRLnW60ugwGujYVnfK9TLKWm1Ys0ZNspy4Wm1Ys0Z7VuWYs0AuWIgfqn0KGTvP_5H00mywhUA7M5HD0UAuW5H00uAPWujYkrRcsnWPAnYc3n17Dn1wjwb7jnWTLwjFDn1bdwW6knLkyiRPmiHwiH7f0IZF9uARqnW0zrH010AFbpyfqwWnkP1n4PHfdnH0LfHR3nHIjPj77rjIanDFaPbuDrDf0UvnqnfKBIjYs0Aq9IZTqn0KEIjYz0AqzTZfqnBnsczYWnH0snan1PjTvPWR3n1R4c10Wna3snj0snj0Wnznknj0sc10WQinsQW0znj0snankQW0snj0snansc10Wna3snj0snj0Wnansc10Wnansc10WnansQW0s0AF9UhV9mvnqnansc10Wn0K3TLwd5HfsrH64P10k0Z7xIWYsQW0sg108njKxna3sn7tsQW0Lg108nWKxna3Ln-tkQWDkg1D8nH7xn7tsg100mMPxTZFEuA-b5H00ThqGuhk9u1Ys0Ad-IjYs0APv5fKGTdqWTADqn0KWTjYs0AN1IjYs0Z7MIvfqn0KETjDqn0KsTjfqn6KWThnqPHbYP1n&us=newvui&xst=TjYzPjDsrHn4n1Ds0ynqnH-anjc1wWParjnkwjnYfYuKf1cLPYfzwjn4PRm3nHPtNb-jyDbYRbkR0ycqwWnkP1n4PHfdnH0LfHR3nHIjPj77rjIanDFaPbuDrDfKT1YkPWnzPHRsnjm4PHbsrjmLn1mvP7tznWNxn07L5UWJs5EDEoy-EebKTHv3l_jekQldGUS40gRqPj04rjbLnjDKIjYkP16srHm4n1f40ydk5H0an0cV0yPC5yuWgLKW0ykd5H0Kmv3qPH0snj0kP1-xpA7EgLKM0gFY5H0KnWf4n1mYPW6LP0&cegduid=Pj04rjbLnjD&solutionId=38965837&word=&ck=1751.13.1780969354557.0.0.161.161.0&shh=www.baidu.com&sht=50000179_hao_pg&wd=) | No | ND | 3 weeks |
| SMITH^[20]^ | 2019 | M/42 | PsO | No | Possible (mother) | Ixekizumab | Yes | CD | Abdominal pain, diarrhea, tenesmus, rectal bleeding, fever | CT,colonoscopy | CRP↑,hypoalbuminemia | No | No | Yes | Infliximab→ustekinumab(psoriasis worsened during infliximab) | No | Remission | 12 weeks |
| DARCH  ^[21]^ | 2020 | F/54 | PsA | No | Yes (sister with UC) | Secukinumab | Yes | CD | Diarrhoea,abdominal discomfort,tenesmus,,weight loss | Colonoscopy | CRP↑,FC↑ | No | No | No | Tildrakizumab | No | Remission | 14 months |
| MU^[14]^ | 2021 | M/45 | PsO | No | No | Ixekizumab | Yes | CD | Abdominal pain, tenesmus,diarrhea | CT,flexible sigmoidoscopy | CRP↑ | Yes,ALT↑,AST↑,ALP↑ | Yes | Yes | None | Total colectomy for toxic megacolon | Remission | 9 months |
| MOROSANU^[13]^ | 2022 | F/42 | PsO、PsA | ND | ND | Ixekizumab | Yes | UC | Abdominal pain, diarrhea, rectal bleeding,  fever | CT,rectosigmoidoscopy | Anemia,WBC↑,CRP↑,hypoalbuminemia | ND | No | Yes | Infliximab | Total colectomy for toxic megacolon | ND | 1 week |
| KHOURI^[22]^ | 2023 | F/38 | PsA | No | No | Secukinumab | Yes | CD | Abdominal pain,diarrhea,hematochezia,oral aphthous ulcerations,odynophagia,weight loss | CT,esophagogastroduodenoscopy,colonoscopy | ESR↑,CRP↑,anemia | ND | ND | Yes | Infliximab | No | Remission | 1 month |
| GRüMME^[23]^ | 2023 | F/35 | SAPHO syndrome | No | No | Secukinumab | Yes | UC | Bloody diarrhea,  abdominal pain,fever,weight loss | Rectosigmoidoscopy | WBC↑,CRP↑,FC↑ | ND | No | Yes | Adalimumab → golimumab (due to autoantibodies) | No | Remission | 3 months |
| GRüMME^[23]^ | 2023 | M/41 | PsA | No | No | Ixekizumab | Yes | Unclassified colitis and ileitis | Bloody stools, abdominal  pain, nausea, tarry  stools | Gastrointestinal endoscopy | CRP↑,FC↑ | Yes，  ALT↑,GGT↑ | Yes | No | Adalimumab | No | Remission | 3 months |
| ZAMPELI^[24]^ | 2024 | M/65 | PsO | No | No | Secukinumab | Yes | CD | Abdominal pain,bloody  diarrhea | CT,flexible sigmoidoscopy,magnetic resonance enterography | Anemia,WBC↑,PLT↑,ESR↑,CRP↑,FC↑ | ND | No | Yes | Certolizumab (ineffective) → risankizumab | No | Remission | Post-induction |
| ISHIDA^[17]^ | 2025 | M/20 | PsO | ND | ND | Secukinumab | Yes | UC | Diarrhea | Colonoscopy | WBC↑,CRP↑,LRG1↑,hypoalbuminemia | ND | No | Yes | Mirikizumab | No | Remission | 4 months |
| ONGAY^[25]^ | 2025 | F/56 | PsA、AS | Yes | ND | Secukinumab | Yes | UC | Bloody diarrhea,weight loss | CT,sigmoidoscopy | Anemia,CRP↑,FC↑,hypoalbuminemia | ND | No | Yes | Risankizumab | No | Remission | 3 months |
| GELDOF^[26]^ | 2025 | M/31 | PsA | ND | ND | Secukinumab | Yes | UC | Abdominal pain, bloody diarrhoea,  weight loss,fever | ultrasound,sigmoidoscopy | CRP↑ | ND | No | Yes | Infliximab → ustekinumab (psoriasis relapse, infliximab-induced hepatitis and arthritis) | No | Remission | ND |
| Present Pt | 2026 | M/54 | PsA | No | No | Vunakizumab | Yes | UC | Abdominal pain, diarrhea, bloody purulent stool, fever | CT,Colonoscopy | WBC↑,CRP↑,ESR↑,anemia,hypoalbuminemia | Yes,ALT↑,AST↑,GGT↑ | No | Yes | Guselkumab | No | Remission | 5weeks |

IBD, inflammatory bowel disease; IL-17i, interleukin-17 inhibitor; F, female; PsO, psoriasis; ND, not documented; CD, Crohn’s disease; WBC, white blood cell; CRP, C-reactive protein; UC, ulcerative colitis; ALB, albumin; HB, hemoglobin; M, male; PsA, psoriatic arthritis; FC, fecal calprotectin; ALT, alanine transaminase; AST, aspartate transaminase; ALP, alkaline phosphatase; ESR, erythrocyte sedimentation rate; GGT, gamma-glutamyl transferase; PLT, platelet; LRG1, leucine-rich alpha-2-glycoprotein 1; AS, ankylosing spondylitis; ND, not documented; Pt, patient.
